# Long-Term Influence of Incidental Emotions on the Emotional Judgment of Neutral Faces

**DOI:** 10.3389/fpsyg.2021.772916

**Published:** 2022-01-06

**Authors:** Marta F. Nudelman, Liana C. L. Portugal, Izabela Mocaiber, Isabel A. David, Beatriz S. Rodolpho, Mirtes G. Pereira, Leticia de Oliveira

**Affiliations:** ^1^Laboratory of Neurophysiology of Behaviour, Department of Physiology and Pharmacology, Biomedical Institute, Universidade Federal Fluminense, Niterói, Brazil; ^2^Department of Physiological Sciences, Biomedical Center, Roberto Alcantara Gomes Biology Institute, Universidade do Estado do Rio de Janeiro, Rio de Janeiro, Brazil; ^3^Laboratory of Cognitive Psychophysiology, Department of Natural Sciences, Institute of Humanities and Health, Universidade Federal Fluminense, Rio das Ostras, Brazil

**Keywords:** incidental, neutral faces, modulation, behavior, emotion

## Abstract

**Background:** Evidence indicates that the processing of facial stimuli may be influenced by incidental factors, and these influences are particularly powerful when facial expressions are ambiguous, such as neutral faces. However, limited research investigated whether emotional contextual information presented in a preceding and unrelated experiment could be pervasively carried over to another experiment to modulate neutral face processing.

**Objective:** The present study aims to investigate whether an emotional text presented in a first experiment could generate negative emotion toward neutral faces in a second experiment unrelated to the previous experiment.

**Methods:** Ninety-nine students (all women) were randomly assigned to read and evaluate a negative text (negative context) or a neutral text (neutral text) in the first experiment. In the subsequent second experiment, the participants performed the following two tasks: (1) an attentional task in which neutral faces were presented as distractors and (2) a task involving the emotional judgment of neutral faces.

**Results:** The results show that compared to the neutral context, in the negative context, the participants rated more faces as negative. No significant result was found in the attentional task.

**Conclusion:** Our study demonstrates that incidental emotional information available in a previous experiment can increase participants’ propensity to interpret neutral faces as more negative when emotional information is directly evaluated. Therefore, the present study adds important evidence to the literature suggesting that our behavior and actions are modulated by previous information in an incidental or low perceived way similar to what occurs in everyday life, thereby modulating our judgments and emotions.

## Introduction

When studying for a test, an individual may incidentally receive an unrelated message providing bad news. Although this event is incidental and unrelated to the individual’s current goals, growing evidence from the literature indicates that incidental events can influence our emotions and behaviors without our intentional effort (Loewenstein and Lerner, 2003; Berkman and Lieberman, 2009). Indeed, it is well documented that emotional processing is extremely influenced by incidental events as different types of contextual factors can modulate behavior, electrocortical activity, and physiological and neural responses during the viewing of emotional pictures (Foti and Hajcak, 2008; Mocaiber et al., 2009, 2010, 2011; Oliveira et al., 2009). For instance, antecedent descriptions stating that pictures of mutilated bodies presented in an experiment were taken from movie productions can attenuate the emotional impact of unpleasant pictures (Mocaiber et al., 2009, 2010, 2011; Oliveira et al., 2009). In addition, some studies have found that incidental negative emotions or affective states can pervasively carry over from one event to next, affecting fairness decisions (Liu et al., 2016), cognitive capacity (Figueira et al., 2017) and emotion recognition (Qiao-Tasserit et al., 2017).

Importantly, the ability to identify emotional expressions can be considered an adaptive social behavior as emotional perception can influence the production and regulation of affective states (Bourke et al., 2010). Accumulating evidence from the literature indicates that facial stimuli may be subject to influences from contextual factors (de Gelder et al., 2006; Aviezer et al., 2008, 2011; Barrett et al., 2011; Wieser and Brosch, 2012), probably because facial expressions are typically embedded in a rich context in real life (Aviezer et al., 2011). Furthermore, facial expressions are extremely important for social interaction as they provide information about other people’s emotions and social intentions (Wieser and Brosch, 2012). It is also worth noting that different types of contexts are able to influence the perception and evaluation of faces (Kim et al., 2004; Aviezer et al., 2008; Righart and De Gelder, 2008).

Contextual information is particularly powerful when facial expressions are either ambiguous or unrelated to any specific emotion, such as neutral faces (Ekman and Friesen, 1976; Lee et al., 2008). Other recent evidence showing that contextual cues can influence our evaluation of neutral faces was obtained from studies using an original behavioral paradigm based on a version of the filmic “Kuleshov effect” (e.g., Pudovkin, 1970; Kuleshov, 1974; Barratt et al., 2016; Calbi et al., 2017, 2019). Furthermore, recent studies have demonstrated that the social context in which a neutral face is presented can impact participants’ emotional responses to such faces (Davis et al., 2010; Schwarz et al., 2013; Wieser et al., 2014; Klein et al., 2015; McCrackin and Itier, 2018). Such contextual modulation of neutral faces has been observed in studies using behavioral paradigms and electrocortical and neuroimaging studies (Davis et al., 2010; Schwarz et al., 2013; Suess et al., 2015).

For example, in a social conditioning study, Davis et al. (2010) demonstrated that neutral faces conditioned with positive and negative context cues subsequently provoked higher activations in the lateral ventral amygdala. Studying the contextual modulation of neutral faces can be especially important because the response to these faces can help to identify mental disorders (Leppänen et al., 2004; Jiao et al., 2011; Oliveira et al., 2013; Wieser and Moscovitch, 2015; Liu et al., 2016; Manelis et al., 2019).

However, little research has investigated whether emotional contextual information presented during a preceding and unrelated experiment could pervasively carry over from one experiment to the next to modulate neutral face processing. Notably, Pichon et al. (2012) demonstrated that prior exposure to negative words led to increased activation in the basal amygdala in a subsequent, unrelated visual task. It remains unknown, however, whether negative contextual information presented during a preceding and unrelated experiment could modulate the emotional judgment of *neutral faces* in a subsequent experiment. In the current study, we assessed whether an emotional text presented in a first experiment could attribute negative emotion to neutral faces in a second experiment. Specifically, our sample comprised two groups: in one group, participants read a neutral text about the loss of an identity card, and in the other group, participants read a negative text about sexual abuse. Subsequently, the participants performed a second experiment with two tasks unrelated to the previous task. The attentional task consisted of judging the orientation of two peripheral bars while pictures (neutral faces or objects) were presented as distractors, and the emotional judgment task consisted of judging the valence of the pictures (neutral faces or objects) while bars were presented as distractors. We predicted that negative text presented previously in the first experiment would modulate both subsequent tasks in the second experiment when compared to neutral text. Therefore, it was expected that the present study could contribute to elucidating the role of long-term modulation of incidental cues in our behavior and actions. This study is important because it highlights that incidental information may have the potential to implicitly change our judgments and emotions, directly affecting our everyday lives.

## Materials and Methods

### Participants

One hundred and thirteen students at Fluminense Federal University participated in this study. Participants were excluded from the analyses for excessive behavioral errors, i.e., incorrect key responses in the bar judgment (>20%, *n* = 11), for failing to fully complete the questionnaire battery (*n* = 1) and for data acquisition problems (*n* = 2). The final sample consisted of ninety-nine subjects (all women) with a mean age of 21.6 s.d 4.3 years (18 years old-51 years old). In the final sample, fifty-five participants were in the negative context and forty-four participants were in the neutral context. We only tested women to avoid wide variations in the emotional impact of the earlier text presented in the first experiment (see Supplementary Material for details regarding the sample characteristics).

### Apparatus and Stimuli

Participants were tested in a sound-attenuated room under dim ambient light. Eprime v2.0 software (E-PrimeVR software, Psychology Software Tools Inc., Pittsburgh, PA, United States) was used to control the stimulus timing and presentation as well as for the collection of responses. The participants positioned themselves on a head-and-chin rest 57 cm from the screen.

Two classes of pictures (45 neutral faces and 45 neutral objects) were employed in the behavioral experiment. All faces were photographs of a man with a neutral expression, each photograph had a different identity, 16 photographs were obtained from Karolinska Directed Emotional Faces (KDEF) (Goeleven et al., 2008), and 29 photographs were obtained from Dallas DataBase (Minear and Park, 2004), which are research databases of human facial expressions containing a valid set of affective and neutral facial pictures. The objects were obtained from the World Wide Web and consisted of photographs of daily life objects. All pictures were presented in grayscale (Supplementary Material).

Pictures depicting faces and objects were matched in terms of brightness, contrast, and spatial frequency to avoid confounding effects unrelated to our purpose. The neutral face and object pictures did not differ with regard to brightness [*t*(59) = 0.54; *P* = 0.60], contrast [*t*(59) = 0.32; *P* = 0.27], or spatial frequency [*t*(59) = 0.19; *P* = 0.84] according to the procedure described by Bradley et al. (2007).

### Design and Procedure

**FIGURE 1:**
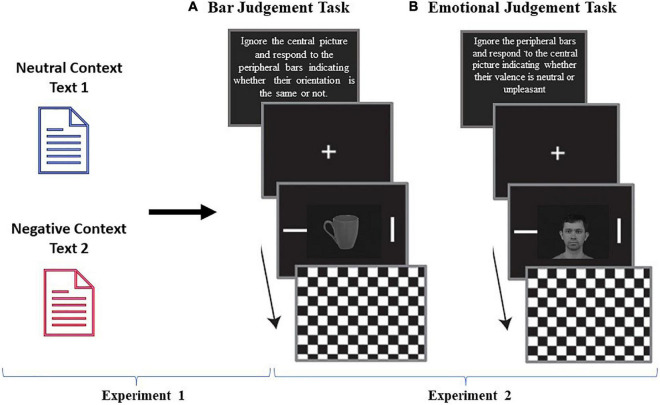
Schematic representation of the sequence of events in each task. During the text rating task experiment, participants read a text and rated the text on a 1–9 scale in terms of valence and arousal (SAM). The subsequent behavioral experiment consisted of two judgment tasks: emotional judgment and bar orientation judgment. The central pictures could be neutral faces (45) or neutral objects (45) and were presented once in each task. Each trial began with a fixation cross that was presented for 1,500 ms, which was followed by a central picture and two peripheral bars that were presented for 200 ms. A checkerboard mask then appeared and remained on the screen until the participant gave a response or the maximum amount of allotted time (1,500 ms) passed. In the bar judgment task **(A)**, participants were instructed to ignore the task-irrelevant central pictures and to respond as quickly and accurately as possible regarding the orientation of the peripheral bars by indicating whether the orientation of the bars was the same. In the emotional judgment task **(B)**, participants were instructed to ignore the peripheral bars and respond if they judged the central picture as neutral or negative. The order of the type of task, bar judgment or emotional judgment was randomized across the subjects. Facial image is from Karolinska Directed Emotional Faces (KDEF) database number AM04NES.

The experimental session consisted of two independent experiments, a “text rating” experiment and a “behavioral” experiment, conducted on the same day. Participants were told they had to perform two independent and unrelated experiments on the same day (Figure 1). The first experiment, the “text rating task,” consisted of reading and rating a negative or a neutral text (Supplementary Material). Participants were randomly assigned to the negative or neutral text group. The second experiment, i.e., the “behavioral” experiment, consisted of the following two judgment tasks: (1) a bar orientation judgment of two peripheral bars and (2) emotional judgment of a picture presented at the center of the screen. The central pictures could be neutral faces or neutral objects. Neutral object pictures were included to serve as a baseline for general effects of mood induction.

At the beginning of the first experiment, the participants read a negative or neutral text depending on the group to which they were assigned. The neutral text discussed the loss of an identity card. The negative text discussed child sexual abuse. The texts were matched regarding the number of phrases, verbs, verbal locations, pronouns, substantives, adjectives, and words. After reading the text, participants were asked to rate the extent to which the text emotionally impacted them by using the paper-and-pencil version of the SAM (Self-Assessment Manikin) scale (Bradley and Lang, 1994). They rated the text on a 1–9 scale in terms of valence (from negative to positive) and arousal (from low to high). There was no time limit to complete the SAM task, but on average, the participants took approximately 10 min to complete this task.

After finishing the text rating part, participants were informed that they would participate in a second experiment that was unrelated to the first one. The interval between the experiments was approximately 5 min. In this subsequent behavioral experiment, participants performed two judgment tasks, an emotional judgment task in which they had to decide whether the presented central picture was neutral or unpleasant and a bar orientation judgment task in which they had to decide whether two peripheral bars had the same orientation. Note that in the bar orientation task, the central picture was task-irrelevant and acted as a distractor. We selected these two tasks to evaluate whether the incidental text reading experiment would impact the participants’ behavior in a subsequent and unrelated experiment in two different aspects: their declarative judgment about the valence of neutral stimuli (emotional judgment task) and the ability of neutral distractive faces or objects to grab attention and interfere in the performance of the main task (bar orientation task). Both the emotional and the bar orientation judgment tasks were conducted according to Erthal et al. (2005). Each trial began with a fixation cross, which was displayed for 1,500 (± 200) ms. Next, a central picture (9° × 12°) and two peripheral bars (0.3° × 3.0°) were presented for 200 ms. The bars were situated at 9° to the right and left of the center of the picture. A whole-screen black and gray checkerboard mask was then shown and remained on the screen until the participant responded or for a maximum of 1,500 ms. In the bar judgment task, the participants were instructed to ignore the task-irrelevant central picture and to respond as quickly and accurately as possible to the peripheral bars to indicate whether their orientation was the same by pressing a key on the keyboard. Key presses (using the right or left index finger) corresponding to the same/different orientations were counterbalanced across subjects. The angular difference of the bars in non-matching trials was 90°, and each block contained the same number of matching and non-matching trials. In the emotional judgment task, the participants were instructed to ignore the task-irrelevant peripheral bars and to respond as quickly and accurately as possible to the central picture, indicating whether their valence was neutral or unpleasant. The key presses (using the right or left index finger) corresponding to neutral/unpleasant judgments on both tasks were counterbalanced across participants. In the second experiment, all participants performed two tasks, bar judgment and emotional judgment, in a blocked way. Each task consisted of 90 trials. The order of the type of task, bar judgment or emotional judgment, was randomized between subjects. Each task contained the same number of neutral faces (45) and objects (45), and the order of these stimuli (faces or objects) within a task was randomized. The bar judgment task had the same number of match and non-match trials. Before each task, participants performed a practice task containing 24 trials; all pictures were neutral pictures of objects and landscapes from the IAPS (Lang et al., 2005). During these training tasks only, subjects received feedback on the screen. The feedback indicated anticipatory responses (RTs less than 100 ms), slow responses (RTs greater than 1,500 ms), and incorrect key responses (only for practice task prior to the bar judgment task) as well as whether an incorrect key was pressed, but only for the bar judgment task during training and the RTs for correct trials. The training tasks were the only tasks in which feedback was given; it was not included in the analyses. There was a brief rest interval (2–3 min) between emotional judgment and bar judgment. See Figure 1 for an illustration of the experimental design.

See Supplementary Material for more information about the objects and a full version of the incidental text.

### Data Analysis

We performed a Kolmogorov-Smirnov test to evaluate the normal distribution of the data. As the data from the emotional judgment task were not normally distributed, we chose to apply a non-parametric approach in all analyses performed in this study, i.e., judgment of the text in the first experiment and the bar judgment task and emotional judgment task analyses in the second experiment.

#### Judgment of the Text in the First Experiment

Valence and arousal ratings attributed to each text were averaged across the participants. The difference between each type of text was compared using a Mann-Whitney test. The alpha level for statistical significance in this analysis was *P* ≤ 0.05.

#### Bar Judgment Task in the Second Experiment

We analyzed the reaction time for the bar judgment task. All anticipatory, slow, multiple or incorrect responses were excluded from further analysis. The mean RT for the bar judgment task when neutral faces and objects were presented as distractors was calculated for each participant. We then created an index for each context (neutral and negative context) by subtracting the mean response time for the bar judgment task when objects were distractors from those obtained for face trials as distractors. Positive values of the index indicate that participants were slower for neutral face stimuli as distractors than for object stimuli as distractors, and negative values indicate that participants were faster for neutral face stimuli than for object stimuli. To investigate whether the attribution of negative emotion to neutral faces changes the reaction time in the bar judgment task, we used these indices (faces-objects) and performed a Mann-Whitney test to compare the bar judgment task between the two contexts. For the analyses, the alpha level for statistical significance was *P* ≤ 0.05.

#### Emotional Judgment Task in the Second Experiment

For the emotional judgment task, we calculated the number of faces and objects rated as negative for each participant in the negative and neutral contexts. Slow, multiple and anticipatory responses were excluded from further analysis. The median and IQR of the faces and objects in each condition are shown in Table 1.

**TABLE 1:** Median and interquartile range (IQR) of faces and objects rated as negative in each context.

Stimulus	Context	Median	IQR
Face	Negative	20.00	4.0–45.0%
Object	Negative	0.00	0.0–15.0%
Face	Neutral	12.50	3.25–44.0%
Object	Neutral	1.00	0.0–41.0%

Additionally, we calculated an index for each context (neutral and negative context) by subtracting the number of objects rated as negative from the number of faces rated as negative. Positive values indicate that more faces were rated as negative than objects, and negative values indicate that more objects were rated as negative than faces. To investigate whether the attribution of negative emotion to neutral faces increases the number of faces judged as negative in the emotional judgment task, we used these indices (faces-objects) and performed a Mann-Whitney test between the two contexts. The alpha level for statistical significance was *P* ≤ 0.05.

See Supplementary Material for additional analysis of the data.

## Results

### Judgment of the Text in the First Experiment

The mean values obtained for valence and arousal ratings and the results of the Mann-Whitney test are described in Table 2. As expected, the negative text was rated as more unpleasant (*U* = 38.50, *P* < 0.0001) and arousing (*U* = 170, *P* < 0.0001) than the neutral text.

**TABLE 2:** Median and interquartile range (IQR) of valence and arousal of each context.

Dimension	Context	Median	IQR	Mann-Whitney
Valence	Negative	1.0	1.0–2.0%	<0.0001
Valence	Neutral	5.0	5.0–5.0%	
Arousal	Negative	8.0	7.0–9.0%	<0.0001
Arousal	Neutral	3.0	1.25–3.0%	

### Bar Judgment Task in the Second Experiment

**FIGURE 2:**
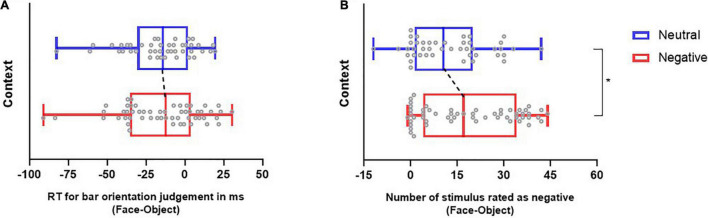
The impact of context on emotional judgment and bar judgment tasks. Reaction time in the bar judgment task. **(A)** Values represent the bar judgment index (face-object RT) per context. **(B)** Quantification of stimulus judged as negative. Values represent the emotional judgment index (face-object) per context. Note that we found an increase in neutral faces judged as negative in the negative context in comparison to the neutral context. Boxplots present the median, the interquartile range and the minimum and maximum values. The circles represent the data for each subject per condition, the black dashed line marks the shift between medians, and *P ≤ 0.05.

We compared the indices (subtraction of mean response time for bar judgment task when objects were distractors from those when faces were distractors) of the reaction time obtained for each context to test whether the indices obtained were significantly different from each other. The results of the Mann-Whitney test demonstrated that the RT index obtained in the negative context was not significantly different from that obtained in the neutral context (*U* = 1164; *p* = 0.74). In the negative context, the median was −12.24, (Q1 = −35.38; Q3 = 3.96), and in the neutral context, the median was −14.08 (Q1 = −30.51; Q3 = − 1.99) (see Figure 2A).

### Emotional Judgment Task in the Second Experiment

The indices (subtraction of number of objects rated as negative from the number of faces rated as negative) obtained during the neutral context and the negative context are illustrated in Figure 2B. We compared the judgment index obtained in each context to test whether the indices were significantly different from each other. The results of the Mann-Whitney test demonstrated that the index obtained in the negative context was significantly different from that obtained in the neutral context (*U* = 936.5; *P* = 0.05). In the negative context, the median was 17.0 (Q1 = 4.0; Q3 = 34.0), and in the neutral context, the median was 10.5 (Q1 = 1.25; Q3 20.0). These results indicate that participants rated more faces as negative in the negative context than in the neutral context. This suggests the effectiveness of the negative modulation by the negative text.

## Discussion

The main goal of the present study was to assess whether the emotional text presented in the first experiment could generate a negative bias in the processing of faces presented in the second and unrelated experiment using the following two tasks: (1) an attentional task in which neutral faces were presented as distractors and (2) a neutral face emotional judgment task. As expected, we found that negative text was judged as more unpleasant and arousing than neutral text, showing that our manipulation to induce a negative emotion in the first experiment was successful. Importantly, our results showed that the judgment index in the second experiment after reading negative text was significantly higher than that obtained after reading neutral text, indicating that participants rated more faces as negative in the negative context than in the neutral context. To the best of our knowledge, this is the first study to demonstrate that emotion information available in a previous experiment that was unrelated to the current goal could increase participants’ propensity to interpret neutral faces in a more negative way. However, one unexpected aspect of our findings is that for the bar judgment task, the reaction time index obtained in the negative context was not significantly different from that obtained in the neutral context. Therefore, our results further demonstrate that the attribution of emotion to neutral faces occurred only in the emotional judgment task.

Our findings are in line with previous literature indicating that neutral face stimuli can be subject to influences from contextual factors (Anderson et al., 2011; Suess et al., 2015; Wieser and Moscovitch, 2015; Zhao et al., 2017; Baum and Abdel Rahman, 2021). Moreover, these results add to the literature by showing that negative contextual information presented in a preceding and unrelated experiment could attribute emotion to neutral faces in a subsequent experiment with ∼12 min of duration and an interval of a few minutes (approximately 5 min) between the experiments. Our findings counter the idea that emotional context effects dissipate shortly after exposition, as in paradigms where emotional primes (e.g., words) are presented for durations shorter than a few hundred milliseconds (Hermans et al., 2001). Consistent with our findings, Qiao-Tasserit et al. (2017) show that a sustained transient emotional state induced by negative emotional clips outlasts the exposure period by ∼2 min and subsequently biases the interpretation of morphed facial expressions ranging from fear to happiness in a forced-choice emotion classification task. In fact, induced negative states increase participants’ propensity to modulate the interpretation of ambiguous expressions relative to induced neutral states. We further extend previous work by demonstrating that (1) we can attribute negative emotion that pervasively carries over from one experiment to the next, (2) the effect in the emotional judgment task is observed between groups, and (3) negative text generates incidental negative emotions that outlast the exposure period by at least ∼8 min (when the emotional judgment task was present first) and a maximum of 15 min.

In line with the idea that emotional context can have long-term effects after exposition, Pichon et al. (2012) found that prior exposure to negative words (relative to neutral words) modifies attention control in a subsequent, unrelated visual task. Our work differs from Pichon et al. (2012) as instead of asking the participants to match (same/different judgment) the stimulus pair at attended locations in an attention task, we used an emotional judgment in which the participants indicated whether the neutral face stimuli valence was neutral or unpleasant.

Notably, only females were chosen as participants in this study. This choice was made because some studies in the literature suggest that an interaction exists between gender and emotional perceptions of faces. For example, some studies have shown that women are better than men in distinguishing facial expressions (Thayer and Johnson, 2000; Guillem and Mograss, 2005). An interaction effect on memory, recognition, and judgment bias also exists between the gender of the face observed and the gender of the participants (Herlitz and Lovén, 2013; Wang, 2013; Harris et al., 2016). For instance, Hofmann et al. (2006) and Hoffmann et al. (2010) showed that women were more accurate than men in recognizing subtle facial displays of emotion. Another important issue is that we used text discussing sexual abuse in this study. In general, females are more likely to be victims of sexual violence than males (de Waal et al., 2017). Therefore, it is expected that the impact of this type of text is greater among females than males, even among females who have not suffered from this traumatic event.

We found an unexpected but interesting result for the bar orientation task: the reaction time index obtained in the negative context was not significantly different from that obtained in the neutral context. Similar to our study, Qiao-Tasserit et al. (2017) found that induced negative states bias the interpretation of facial expressions in perceptual decision-making tasks, but the reaction time measurements for this paradigm lacked the sensitivity necessary to detect this effect. An explanation may be that the reaction time is a more implicit measure compared to the emotional judgment of a neutral face for directly accessing the attribution of emotion generated by the preceding text. Therefore, cognitively assessed affective content can be more easily modulated by incidental information than less cognitive emotional content, such as that measured by reaction time. This notion is consistent with previous studies showing that participants rated neutral faces as negative significantly more in negative contexts (fearful and sadness) than in other contexts (neutral or happy/desire contexts) (Barratt et al., 2016; Calbi et al., 2017). However, more implicit measures, such as eye tracking, did not support the expected differences between the context conditions (Barratt et al., 2016). Furthermore, Deveney and Pizzagalli (2008) showed that their participants regulated their emotions to unpleasant pictures regardless of whether the word was negative or neutral, whereas their reaction time data failed to show emotion regulation modulation.

There were also some limitations in the present study. The main limitation was that after the experiment, we did not directly assess whether any participant guessed that the experiments were linked.

In summary, the present work adds to the literature important evidence that our behaviors and actions are modulated by preceding information in an incidental way. Previous negative information generated by reading a text was able to attribute emotion to neutral faces in a subsequent experiment presented several minutes later. These results promote reflection on how much deliberate and conscious control we have over our decisions, actions, and attitudes in our everyday life.

## Data Availability Statement

The original contributions presented in the study are included in the article/Supplementary Material, further inquiries can be directed to the corresponding author/s.

## Ethics Statement

The studies involving human participants were reviewed and approved by the Research Ethics Committee of Fluminense Federal University. The patients/participants provided their written informed consent to participate in this study.

## Author Contributions

MN analyzed and interpreted the data, wrote the manuscript, and contributed to the data organization. LP contributed to the data organization, data interpretation, and revising the manuscript. IM, ID, and BR interpreted the data and revised the manuscript. MP contributed to writing and revising the manuscript and the data interpretation. LO analyzed, interpreted the data, wrote, and revised the manuscript. All authors read and approved the final manuscript.

## Conflict of Interest

The authors declare that the research was conducted in the absence of any commercial or financial relationships that could be construed as a potential conflict of interest.

## Publisher’s Note

All claims expressed in this article are solely those of the authors and do not necessarily represent those of their affiliated organizations, or those of the publisher, the editors and the reviewers. Any product that may be evaluated in this article, or claim that may be made by its manufacturer, is not guaranteed or endorsed by the publisher.
