# Supplementary material for: Long-Term Influence of Incidental Emotions on the Emotional Judgment of Neutral Faces
Source: Front Psychol. 2022 Jan 6;12:772916. doi: 10.3389/fpsyg.2021.772916 (PMC8773088; doi:10.3389/fpsyg.2021.772916)
Supplement: Supplementary file 1 [file Data_Sheet_1.docx]

**Supplemental Material - Long-term influence of incidental emotions on the emotional judgement of neutral faces**

**Methods**

**Participants**

All participants were undergraduate students of the Federal Fluminense University, Niteroi, Brazil, reported no history of neurological or psychiatric disorders, had normal or corrected-to-normal vision, were not taking any medications acting on the central nervous system and reported no history of sexual abuse. Participants with previous sexual abuse experience were not included as such experience might impact facial recognition and has been associated with emotional disorders (Roberts et al., 2020; Amado, et al.,. 2015; Islam‐Zwart, et al., 2005). The presence of a history of sexual abuse was assessed using the Trauma History Questionnaire translated and adapted to Portuguese by Fiszman et al. (2005) from the original [Green, 1996] and the Childhood Trauma Questionnaire - Short Form (Bernstein., 1998, 2003; Grassi-Oliveira and Stein, 2008). The subjects were naive to the purpose of the experiment.

**Apparatus and Stimuli**

**List of Objects:**

air conditioning

food mixer

chest

drinking fountain

ball

handbag

notebook

coffee machine

speaker

cardboard box

calculator

mug

basket

hat

pencil sharpener

excavator

pencil case

extender

iron

filter

fridge

bottle

jar

can

blender

megaphone

laptop

bedside lamp

perfume bottle

dustpan

umbrella

pan

brush tool

tire

bowl

dish

radio

saltshaker

couch

telephone

sneakers

bowl

toaster

guitar

cup

**Design and Procedures**

**Texts:**

**Negative Text**

Report 1- J. 15 years

“My uncle sat next to me to watch the video game I played with my cousins. When I saw him, he reached inside the long sleeve of my nightgown. His hand reached my breasts, and he massaged them for a while. A few minutes later, my uncle gave up on my boobs and decided to reach inside my panties. I understood that this situation was very wrong, and I got up to go to the bedroom to sleep. My bed that night would be a mattress on the floor between my cousins' beds. I know I woke up in the middle of the night with him on top of me, trying to touch me in every way again. His breath smelled of alcohol."

Report 2 - L. 14 years old

“When I was 3 years old, my mother had severe depression. My older brother took over the house and took care of me and our younger brother. I learned many years later that at this time, my mother had caught him doing oral sex and touching my genitals while he bathed me. She threatened to throw him out of the house. But, without family support and without strength, he decided to leave things as they were as he needed to support us. At the age of 8, my brother took my virginity. I remember that something strange happened, that I was hurt, but since I was young, the memories faded a little. The harassment continued until I was 12 years old.”

Similar to these cases, there are thousands of cases of pedophilia in which victims suffer from abuse for long periods of their lives. Brazil is one of the countries with the highest number of pedophiles worldwide with 5.4 million Brazilians reporting sexual abuse in childhood (Unifesp, 2014). The police receive approximately 70 allegations of pedophilia a day and have already cataloged several criminals.

**Neutral Text**

Report 1- R. 18 years old

“Whenever I had my team's game on the weekend, I would go to my cousins' house and spend the whole weekend there. The stadium was close to their house; so, we all went together. Once, when I got home, I realized that I had lost my ID card, probably during the departure. We searched all the way, but we didn't find it. Because of that, I had to make a duplicate.”

Report 2- P. 21 years old

“When I was 18 years old, I went traveling with my friends to watch a football match of our team. My parents took me to the bus station. Before the trip, I separated all my documents and put them in a separate wallet. When I went to take the bus back home, I realized that I had no ID card. I called the stadium, but they couldn't find my ID in lost and found. Upon returning from my trip, I provided a duplicate. A few years later, the same group of friends decided to schedule another trip to the stadium. This time, it was in our own city. I decided to take only my ID card and some money in my pocket so I wouldn't take the whole wallet. However, when I got home, I couldn't find my ID. I believe it must have fallen while jumping, and I didn't notice. So, at age 25, I had to make another copy of my identity card for the second time.”

Similar to these cases, there are thousands of cases of the loss of an identity card (RG – General Registry) in stadiums. DETRAN reports that it is common to request a duplicate of an identity document more than once due to this type of loss. A catalog was made with the identity cards lost in stadiums.

**Analysis**

**Sample Size Calculation:**

We conducted a pilot study (total sample = 44; 22 per group) to estimate the magnitude of the effect sizes of interest, which was subsequently used in an ‘a priori sample size calculation’ conducted using G*Power 3.1.

**Emotional Judgment Task:**

To estimate the effect size of the difference between the groups, first, we created an index for each context (neutral and negative contexts) by subtracting the number of objects rated as negative (baseline condition) from the number of faces rated as negative. The data from the pilot study revealed an effect size d = 0.70. To detect an effect size of Cohen’s d = 0.70 with 80% power (alpha = .05, two-tailed), G*Power suggests that we need 34 participants per group (N = 68) in a Mann Whitney test (two-groups).

**Bar Judgement Task:**

For the bar judgement task, we also created an index for each context (by subtracting the mean response time to the bar when the objects were distractors from those obtained in the face trials as distractors). The smallest effect size of interest was set to d = 0.29 based on the data obtained in our pilot study (n=44; 22 per group). To detect an effect size of Cohen’s d = 0.29 in a Mann Whitney test (two-groups), G*Power suggests that we need 368 participants to achieve 80% power (alpha = .05, two-tailed).

**Results**

**Reaction Time in the Emotional Judgement Task:**

The reaction data from the emotional judgement task were also analysed by creating an index (subtracting the mean response time in the emotional judgement task to objects from the response times to faces) and testing for differences between the contexts using Mann-Whitney tests. The results showed that there was no significant difference in reaction time between the contexts in the emotional judgement task (U= 1070 p= 0.3266). In the negative context, the median was 9.05 (Q1= -24.40; Q3= 117.7), and in the neutral context, the median was 38.98 (Q1= -11.06 ; Q3 = 300.5). Figure S1 illustrates these results.


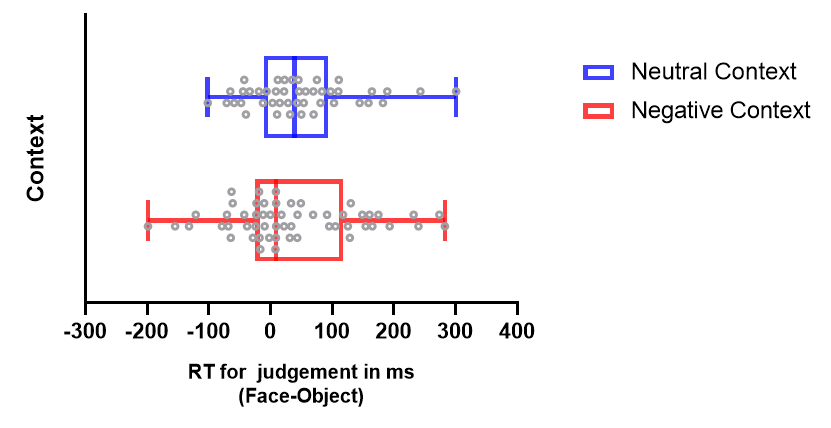


Figure S1: **Impact of context on reaction time in the emotional judgement task**. Values represent the reaction time in the emotional judgement index (face-object RT) per context. Notably, we did not find a significant modification ofreaction time in the negative context in comparison to the neutral context. Boxplots present the median, the interquartile range and the minimum and maximum values. The circles represent the data of each subject per condition, and the black dashed line marks the shift between medians.

**Mixed ANOVA Analysis**

For completeness, we also performed a data analysis using mixed ANOVA models with the “Stimulus category” (Face or Object) as the within-subjects factor and “Context”(Neutral or Negative) as the between-groups factor, followed by a Newman-Keuls post hoc analysis. The results of these analyses of both types of tasks are discussed below.

**Emotional Judgement Task**

We found a main effect of stimulus (F(1, 97) =118.24; p <0.001) and an interaction effect between the context and stimuli (F(1, 97) =4.69; p =0.032). The post hoc tests showed that there is a significant increase in the number of faces judged as negative in the negative context (p=0.01), but there is no difference between the contexts in terms of the object stimuli (p=0.68).

**Bar Judgement Task**

We found only a main effect (stimulus category = (F(1,97) = 38.17; p =0.000); context = ((F(197) = 1.23; p =0.275) but no interaction in this analysis (F(1,97) = 0.038; p = 0.845).

**Error Analysis**

We also conducted a mixed ANOVA analysis to determine whether the errors differed between the contexts in both types of tasks. The results showed that there was only a main effect of stimuli (F(1,97) = 6.54; p= 0.01), (F(1,97)= 7.03; p= 0.009)in the bar judgement and emotional judgement tasks. There was no significant main effect of context or interaction (all p values > 0.46).

**References**

Amado, B. G., Arce, R., and Herraiz, A. (2015). Psychological injury in victimsof child sexual abuse: a meta-analytic review. Psychosoc. Interv. 24, 49–62.doi: 10.1016/j.psi.2015.03.002

Bernstein, D. P., and Fink, L. (1998). Manual for the Childhood TraumaQuestionnaire – A Retrospective Self-Report. New York, NY: Pearson.

Bernstein, D. P., Stein, J. A., Newcomb, M. D., Walker, E., Pogge, D., Ahluvalia,T., et al. (2003). Development and Validation of a brief screening version ofthe Childhood Trauma Questionnaire. Child Abuse Negl. 27, 169–190. doi:10.1016/S0145-2134(02)00541-0

Fiszman, A., Cabizuca, M., Lanfredi, C., and Figueira, I. (2005). The cross-culturaladaptation to Portuguese of the Trauma History Questionnaire to identifytraumatic experiences.

Grassi-Oliveira, R., and Stein, L. M. (2008). Childhood maltreatment associatedwith PTSD and emotional distress in low-income adults: the burden of neglect.Child Abuse Negl. 32, 1089–1094. doi: 10.1016/j.chiabu.2008.05.008

Islam-Zwart, K. A., Heath, N. M., and Vik, P. W. (2005). Facial recognitionperformance of female inmates as a result of sexual assault history. J. Trauma.Stress 18, 263–266. doi: 10.1002/jts.20025

Roberts, A. L., Sumner, J. A., Koenen, K. C., Kubzansky, L. D., Grodstein, F., Rich-Edwards, J., et al. (2020). Childhood abuse and cognitive function in a largecohort of middle-aged women. ChildMaltreat. doi: 10.1177/1077559520970647[Epub ahead of print].
